# Association of immune relevant single nucleotide polymorphisms with ALK-positive anaplastic large cell lymphoma presentation and outcome: results of the immuno ALCL study

**DOI:** 10.1186/s12967-025-07410-5

**Published:** 2025-12-30

**Authors:** Petrazzuolo Adriana, Vincent Carbonnier, Fatima Domenica Elisa De Palma, Maria Perez-Lanzon, Véronique Vergé, Cyril Quivoron, Laurence Lamant, Laurence Brugieres, Charlotte Rigaud, Stéphane Ducassou, Marie-Emilie Dourthe, Marie-Cécile Le Deley, Christine Damm-Welk, Willi Woessmann, Véronique Minard-Colin, Maria Chiara Maiuri, Guido Kroemer

**Affiliations:** 1 International Center for T1D, Pediatric Clinical Research Center Romeo ed Enrica Invernizzi, DIBIC, Università degli Studi di Milano, Milan, Italy; 2 Team “Metabolism, Cancer & Immunity”, Centre de Recherche des Cordeliers UMRS1138, INSERM, Université Paris Cité, Sorbonne Université, Paris, 75006 France; 3 Cell Biology and Metabolomics Platforms, Gustave Roussy Cancer Campus, Villejuif, 94805 France; 4 Department of Molecular Medicine and Medical Biotechnologies, University of Napoli Federico II, Napoli, Italy; 5 Department of Medical Biology and Pathology, Gustave Roussy Cancer Campus, Villejuif, France; 6 Translational Research Hematological Laboratory, Gustave Roussy, Villejuif, France; 7 INSERM U1170, Université Paris-Saclay, Gustave Roussy, Villejuif, France; 8 Pathology Department, Institut Universitaire du Cancer Toulouse Oncopole, CHU de Toulouse, Toulouse, France; 9 Department of Pediatric and Adolescent Oncology, Gustave Roussy, INSERM U1015, Université Paris-Saclay, Villejuif, France; 10 Department of Pediatric Oncology and Hematology, Bordeaux University Hospital, Bordeaux, France; 11 INSERM Unité Mixte de Recherche (UMR)-S1151, Centre National de la Recherche Scientifique UMR-S8253, Institut Necker Enfants Malades, Université Paris Cité, Paris, France; 12 Department of Pediatric Hematology, Assistance Publique- Hôpitaux de Paris, Robert Debré Hospital, Université Paris Cité, Paris, France; 13 Department of Clinical Research and Innovation, Centre Oscar-Lambret, Lille, France; 14 Pediatric Hematology and Oncology and NHL-BFM Study Center, Universtiy Medical Center Hamburg-Eppendorf, Hamburg, Germany; 15 Department of Biology, Institut du Cancer Paris CARPEM, Hôpital Européen Georges Pompidou, AP-HP, Paris, 75015 France

**Keywords:** ALK, ALCL, SNPs, Immunity

## Abstract

**Background:**

Single nucleotide polymorphisms (SNPs) of cancer-immunity relevant genes may decide the extent of tumor immunosurveillance and have clinical significance. The Immuno ALCL trial was designed to investigate whether genetic variability in 13 cancer-immunity relevant genes correlated with clinical features and outcome of anaplastic lymphoma kinase (ALK)-positive anaplastic large cell lymphoma (ALCL) patients.

**Methods:**

One hundred eighty patients were enrolled and genotyped for 14 SNPs. Age at diagnosis, progression-free survival, histological subtype and anti-ALK antibody titer data were collected.

**Results:**

IL10 rs1800872, IL10 rs1800896 and TLR3 rs3775291 variants significantly correlated with age at diagnosis. TLR3 rs3775291 was associated with progression-free survival in a recessive model. Combination of multiple genetic variations showed a trend to associate with post-therapeutic relapse. None of the SNP analyzed associated with histological or clinical parameters.

**Conclusion:**

Despite the low number of patients, our work uncovered potential associations between certain cancer-immunity relevant genes and clinical features of ALK-positive ALCL patients. Associations do not imply causations. However, our work highlighted a possible contribution of the IL10 and TLR3 pathways to ALK-positive ALCL pathogenesis and suggested that several genetic variants in concert may modulate the risk of post-therapeutic relapse.

**Trial registration:**

clinicaltrial.gov NCT02902874. Registered 07 September 2016 https//clinicaltrials.gov/study/NCT02902874.

**Supplementary Information:**

The online version contains supplementary material available at 10.1186/s12967-025-07410-5.

## Background

Tyrosine kinase receptor anaplastic lymphoma kinase (ALK) is physiologically expressed during embryogenesis with a critical role in nervous system development. However, chromosomal rearrangements, especially translocations, cause expression of pathogenic ALK fusion proteins that drive tumor formation and maintenance. For instance, the translocation between chromosomes 2 and 5 (t(2;5)) fuses the nucleophosmin 1 (*NPM1*) gene with *ALK* [1], leading to anaplastic large cell lymphoma (ALCL) [2, 3]. The constitutive active NPM1::ALK tyrosine kinase drives tumor transformation.

Multiple lines of evidence suggest that ALK-expressing tumors are immunogenic. Accordingly, ALK-positive ALCL patients develop anti-ALK antibodies [4] and antibody titer against ALK correlate with the clinical stage of the disease and event-free survival [5, 6]⁠. Moreover, ALK-positive ALCL patients exhibit a high frequency of circulating ALK-specific CD8^+^ T cells with an effector or memory phenotype, which indicates recent activation by antigen recognition [7]. In addition, ALK-positive ALCL patients respond to ALK-derived HLA-DRB1 (human leukocyte antigen DR beta 1 chain)-restricted peptides suggesting tumor recognition by CD4^+^ T cells [8]. These observations argue in favor of T cell- and B cell-mediated recognition of ALK-positive ALCL, which may impact tumor progression and relapse [9]. Finally, current treatments of primary or relapsed/refractory [10, 11] ALK-positive ALCL induce tumor cell death in an immunogenic fashion, thus enhancing the immune-mediated control of tumor growth [12, 13].

Genetic variability, in the form of single nucleotide polymorphisms (SNPs), of cancer-immunity relevant genes may have a prognostic or predictive value and correlate with tumor onset or risk of relapse after treatment. For instance, variants of the well-known immunosuppressive receptors PD-1 and CTLA-4, which blunt tumor immunosurveillance, correlate with the risk of developing cancer [14–17]. Likewise, SNPs in genes involved in the immune-mediated recognition of tumor cells upon their immunogenic death, are associated with disease onset and treatment efficacy. Our group demonstrated that the loss-of-function variant rs867228 of *FPR1* is associated with precocious onset of breast, colorectal, esophageal and head & neck carcinomas [18]. Furthermore, SNP rs4986790 of *TLR4* and rs3751143 of *P2RX7* correlate with the frequency of metastases after surgery in women with breast cancer [19, 20]. Since the progression of ALK-positive ALCL is influenced by therapy-independent and therapy-induced anti-tumor immune response, we investigated whether the genotype of 14 cancer-immunity relevant SNPs might correlate with lymphoma presentation and recurrence, as well as with histological features of ALK-positive ALCL.

## Materials and methods

### Study population

Patients from both French and German centers were included in the study. The French cohort consisted of individuals over 3 years of age enrolled in prospective therapeutic trials for ALK-positive ALCL between 1975 and 2020 [21, 22]. Clinical and biological data were collected from the ALCL99, HM91 and COPAD-IGR databases. Written informed consent was obtained from patients or legal guardians, and assent was provided by minors when appropriate. The German cohort included patients treated with BFM-type regimens in the NHL-BFM 95, ALCL99 and NHL-BFM Registry 2012 studies. All participants had consented to an ethically approved protocol investigating immune responses to ALK, which included genetic testing.

### Study design and procedure

For the French group (*n* = 150), eligible patients were identified in the national ALCL database and confirmed by investigators at each participating Société Française de lutte contre les Cancers et leucémies de l’Enfant et de l’adolescent (SFCE) center. Investigators updated clinical data, verified availability of stored sera, obtained consent from surviving patients and organized blood collection. Samples were processed at Gustave Roussy for peripheral blood mononuclear cells (PBMCs) and serum isolation. The study was approved by relevant ethics committees and national authorities and conducted in accordance with the Declaration of Helsinki. The median time from diagnosis to blood sampling was 2.1 years.

For the German group (*n* = 30), blood samples were obtained from patients aged 14 years or older in complete remission. DNA was extracted from PBMCs after double pseudonymization. Anti-ALK antibodies were assessed at diagnosis. Clinical and biological data were provided through the ALCL99 or NHL-BFM registry. ClinicalTrials.gov ID NCT02902874.

### DNA extraction and genotyping

DNA was extracted from PBMCs using the DNeasy Blood & Tissue Kits (Qiagen, Hilden, Germany) following manufacturer’s instruction. SNP genotyping was performed with TaqMan assays (Thermo Fisher Scientific, Waltham, Massachusetts, USA) and TaqMan genotyping Master Mix (Thermo Fisher Scientific) on the StepOnePlus Real-Time PCR System (Applied Biosystems, Waltham, Massachusetts, USA). Cycling conditions were 60 °C for 30 s, 95 °C for 10 min and 40 cycles of 95 °C for 15 s and 60 °C for 1 min. Allelic discrimination was based on FAM/VIC fluorescence (Table 1).

**Table 1:** List of SNPs IDs and TaqMan assay IDs

Gene	SNP ID	Assay ID
CD274	rs4143815	C__31941235_10
CD86	rs1129055	C___7504226_10
CTLA4	rs231775	C___2415786_20
ECE1	rs1076669	C___2464666_30
FPR1	rs867228	C___3266374_1_
IFNAR1	rs1041868	C___1841019_10
IFNGR2	rs17882748	C__61106388_10
IL10	rs1800872	C___1747363_10
IL10	rs1800896	C___1747360_10
PDCD1	rs2227981	C__57931286_20
TGFB1	rs1800469	C___8708473_10
P2RX7	rs3751143	C__27495274_10
TLR3	rs3775291	C___1731425_10
TLR4	rs4986790	C__11722238_20

### Cell culture and treatment

Human NPM1::ALK-positive anaplastic large cell lymphoma (ALCL) SU-DHL-1 cell line was purchased from the German Collection of Microorganisms and Cell Cultures (DSMZ, Braunschweig, Germany) and cultured in RPMI 1640 supplemented with 100 units/mL of penicillin, 100 µg/mL of streptomycin (Pen/Strep) and 10% fetal bovine serum (FBS). Cells were grown in standard conditions (37 °C and 5% CO2) and routinely checked for mycoplasma contamination. All media and supplements were purchased from Gibco (Waltham, Massachusetts, USA). ALCL cells were treated with the ALK inhibitor ceritinib (CER; Selleck Chemicals, Houston, Texas, USA) at 50 nM for 24–48 h.

### RNA extraction and RT-qPCR

RNA was extracted from PBMCs using the RNeasy plus mini kit (Qiagen) following manufacturer’s instruction. Reverse transcription to cDNA was performed using SuperScript™ IV VILO™ Master Mix (Thermo Fisher Scientific). *TLR3*, *IL10* or *GAPDH* were amplified with specific TaqMan Gene Expression assays (Thermo Fisher Scientific) using the TaqMan Fast Advanced Master Mix (Thermo Fisher Scientific) on the StepOnePlus Real-Time PCR System (Applied Biosystems). Relative expression levels were calculated and 2^(−ΔCt) transformation was used for analysis.

### RNA sequencing

RNA was extracted from cell pellets using the RNeasy plus mini kit (Qiagen) and RNA quality (RNA integrity number or RIN) was determined on the Agilent 2100 Bioanalyzer (Agilent Technologies, Palo Alto, California, USA) prior to library preparation with TruSeq Stranded mRNA kits (Illumina). Libraries were then quantified by using the KAPA Library Quantification Kit for Illumina Libraries (KapaBiosystems, Wilmington, Massachusetts, USA), profiled by using the DNA High Sensitivity LabChip kit and finally sequenced on an Illumina Nextseq 500. Data processing used AOZAN software (ENS Paris) for demultiplexing and controlling quality of raw data, STAR algorithm (version 2.5.2b) and Picard tools (version 2.8.1) for alignment and Featurecount (version Rsubread 1.24.1) for read counts. Raw data have been made publicly available on the repository ArrayExpress (accession number E-MTAB-15866).

### Cytokines and immune checkpoint ligands quantification

After treatment, supernatants were collected, and 20 cytokines and immune checkpoint ligands were quantified using Human Procarta Plex kits (Thermo Fisher Scientific) by following the manufacturer’s instructions.

### Detection of anti-ALK antibodies

Detection of anti-ALK antibodies in patients’ sera was performed as previously described by Ait-Tahar and colleagues [5]. Briefly, COS-1 cells (DSMZ) were transiently transfected with ALK-encoding plasmids, incubated with patient’s serum and stained with a rabbit anti-human IgG-HRP. Titers were defined as the highest serum dilution showing a positive signal [4] with 1/750 as the cutoff [6].

### Statistical analysis

All clinical data were pseudonymized prior to analysis. Age at diagnosis and progression-free survival (PFS) were derived from registry data. Associations with SNP genotypes were tested by using Mann-Whitney and log-rank statistical tests. A score-based system was used to assess the correlation between combinations of SNP and PFS. A score of 0, 1 or 2 was assigned to each SNP genotype based on the association with PFS. Lower scores were assigned to genotypes associated with higher risk of relapsing, higher scores to genotypes that correlated with lower risk of relapsing. Patients were grouped in two classes (low or high score) and Kaplan-Meier estimates were used to plot PFS according to patient score. Additional analyses included Fisher’s exact test, Chi-square, student’s t test and Bootstrap t-test p-value adjusted by the Benjamini-Hochberg procedure, with 10,000 permutations. R and GraphPad Prism v9 were used for analysis.

## Results

### Genotyping

One hundred eighty patients diagnosed with ALK-positive ALCL were enrolled in this study. Patients were genotyped for 14 cancer-immunity relevant SNPs, considered as relevant based on our previous works and literature search [14, 15, 18–20, 23–31]. Frequency in the global population and clinical relevance of SNPs are reported in Table 2 (source www.ncbi.nlm.nih.gov/snp and www.ncbi.nlm.nih.gov/clinvar). The frequency in the study population as well as clinical data according to SNP genotype are presented in Table 3.

**Table 2:** SNP frequency and characteristics

			Clinical impact (Clinvar)	
	Allele frequency in global population	Molecular consequences	Impact	Disease
*CD274* rs4143815	G = 0.73823	Non coding transcript variant	Not reported in ClinVar	
	C = 0.26177			
*CD86* rs1129055	G = 0.726306	Missense variant (Ala310Thr)	Benign	
	A = 0.273694			
*CTLA4* rs231775	A = 0.617783	Missense variant (Thr17Ala)	Risk factor	Hashimoto thyroiditis [32]; Thyroid-associated orbitopathy [33]; Systemic lupus erythematosus [34, 35]; Celiac disease [36, 37]; Type 1 and 2 Diabetes [38–41]
	G = 0.382217		Benign	Autoimmune lymphoproliferative syndrome due to CTLA4 haploinsuffiency
*ECE1* rs1076669	G = 0.935386	Missense variant (Thr341Ile)	Benign	
	A = 0.064614			
*FPR1* rs867228	T = 0.201138	Missense variant (Glu346Ala)	Benign	Gingival disorder
	G = 0.798862			
*IFNAR1* rs1041868	G = 0.812593	Intron variant	Not reported in ClinVar	
	A = 0.187407			
*IFNGR2* rs17882748	T = 0.41776	5 Prime UTR variant	Benign	
	C = 0.58224			
*IL10* rs1800872	T = 0.274262	2KB upstream variant	Risk factor	HIV infection [42]
	G = 0.725738		Benign	Inflammatory bowel disease
			Protective	Graft-versus-host disease [43]
*IL10* rs1800896	T = 0.553450	2KB upstream variant	Not reported in ClinVar	
	C = 0.446550			
*PDCD1* rs2227981	A = 0.42806	Synonymous variant	Benign	
	G = 0.57194			
*P2RX7* rs3751143	A = 0.815741	Missense variant (Glu496Ala)	Not reported in ClinVar	
	C = 0.184259			
*TGFB1* rs1800469	A = 0.319484	2KB upstream variant	Benign	Joubert syndrome, Meckel-Gruber syndrome
	G = 0.680516			
*TLR3* rs3775291	C = 0.723658	Missense variant (Leu412Phe)	Benign	Susceptibility to Herpes simplex encephalitis [44]
	T = 0.276342		Protective	Susceptibility to HIV infection [45]
*TLR4* rs4986790	A = 0.941860	Missense variant Asp299Gly	Protective	Pericementitis [46, 47]
	G = 0.058140			

**Table 3:** Study population genotypes and clinical data

		Genotype frequency	Progression			Median age at diagnosis	
		n/tot (%)	YES n/tot (%)	NO n/tot (%)	Chi-square *P* value	Median	CI 95%
*CD274* rs4143815	GG	102/180 (56.7)	26/102 (25.5)	76/102 (74.5)	0.5268	141	130–156
	GC	59/180 (32.8)	17/59 (28.8)	42/59 (71.2)		141	116–154
	CC	19/180 (10.6)	3/19 (15.8)	16/19 (84.2)		172	122–183
*CD86* rs1129055	GG	92/180 (51.1)	28/92 (30.4)	64/92 (69.6)	0.2997	136	124–152
	GA	75/180 (41.7)	15/75 (20)	60/75 (80)		147	135–163
	AA	13/180 (7.22)	3/13 (23.1)	10/13 (76.9)		145	120–166
*CTLA4* rs231775	AA	85/180 (47.2)	23/85 (27.1)	62/85 (72.9)	0.5166	145	130–154
	AG	75/180 (41.7)	20/75 (26.7)	55/75 (73.3)		137	122–155
	GG	20/180 (11.1)	3/20 (15)	17/20 (85)		158	106–169
*ECE1* rs867228	GG	160/180 (88.9)	40/160 (25)	120/160 (75)	0.4833	143	130–153
	GA	20/180 (11.1)	6/20 (30)	14/20 (70)		138	122–174
*FPR1* rs867228	GG	104/180 (57.8)	27/104 (26)	77/104 (74)	nc	143	134–163
	GT	70/180 (38.9)	18/70 (25.7)	52/70 (74.3)		145	128–155
	TT	6/180 (3.33)	1/6 (16.7)	5/6 (83.3)		105	75.5–140
*IFNAR1* rs1041868	GG	117/180 (65)	33/117 (28.2)	84/117 (71.8)	0.4602	142	129–147
	GA	55/180 (30.6)	12/55 (21.8)	43/55 (78.2)		150	124–174
	AA	8/180 (4.44)	1/8 (12.5)	7/8 (87.5)		122	105–154
*IFNGR2* rs17882748	CC	63/179 (35.2)	21/63 (33.3)	42/63 (66.7)	0.1857	149	130–158
	CT	77/179 (43)	18/77 (23.4)	59/77 (76.6)		142	129–163
	TT	39/179 (21.8)	7/39 (17.9)	32/39 (82.1)		135	106–161
*IL10* rs1800872	GG	91/180 (50.6)	25/91 (27.5)	66/91 (72.5)	0.8330	131	123–142
	GT	80/180 (44.4)	19/80 (23.8)	61/80 (76.2)		150	135–171
	TT	9/180 (5)	2/9 (22.2)	7/9 (77.8)		188	70–199
*IL10* rs1800896	TT	52/180 (28.9)	14/52 (26.9)	38/52 (73.1)	0.9396	150	139–178
	TC	94/180 (52.2)	24/94 (25.5)	70/94 (74.5)		133	122–153
	CC	34/180 (18.9)	8/34 (23.5)	26/34 (76.5)		141	120–160
*PDCD1* rs2227981	GG	48/180 (26.7)	14/48 (29.2)	34/48 (70.8)	0.6196	130	112–158
	GA	101/180 (56.1)	26/101 (25.7)	75/101 (74.3)		145	135–157
	AA	31/180 (17.2)	6/31 (19.4)	25/31 (80.6)		128	112–173
*P2RX7* rs3751143	AA	105/180 (58.3)	30/105 (28.6)	75/105 (71.4)	nc	142	129–155
	AC	72/180 (40)	16/72 (22.2)	56/72 (77.8)		146	131–163
	CC	3/180 (1.67)	0/3 (0)	3/3 (100)		91	79–187
*TGFB1* rs1800469	GG	82/180 (45.6)	23/82 (28)	59/82 (72)	0.7808	148	128–161
	GA	72/180 (40)	17/72 (23.6)	55/72 (76.4)		142	132–161
	AA	26/180 (14.4)	6/26 (23.1)	20/26 (76.9)		138	98–163
*TLR3* rs3775291	CC	92/180 (51.1)	23/92 (25)	69/92 (75)	0.1342	146	130–161
	CT	70/180 (38.9)	15/70 (21.4)	55/70 (78.6)		146	134–168
	TT	18/180 (10)	8/18 (44.4)	10/18 (55.6)		99	68–130
*TLR4* rs4986790	AA	165/180 (91.7)	43/165 (26.1)	122/165 (73.9)	0.5152	142	129–150
	AG	15/180 (8.33)	3/15 (20)	12/15 (80)		141	61–163

nc: criteria for Chi-square test were not met

### Association of polymorphisms with age at diagnosis

**Fig. 1:**
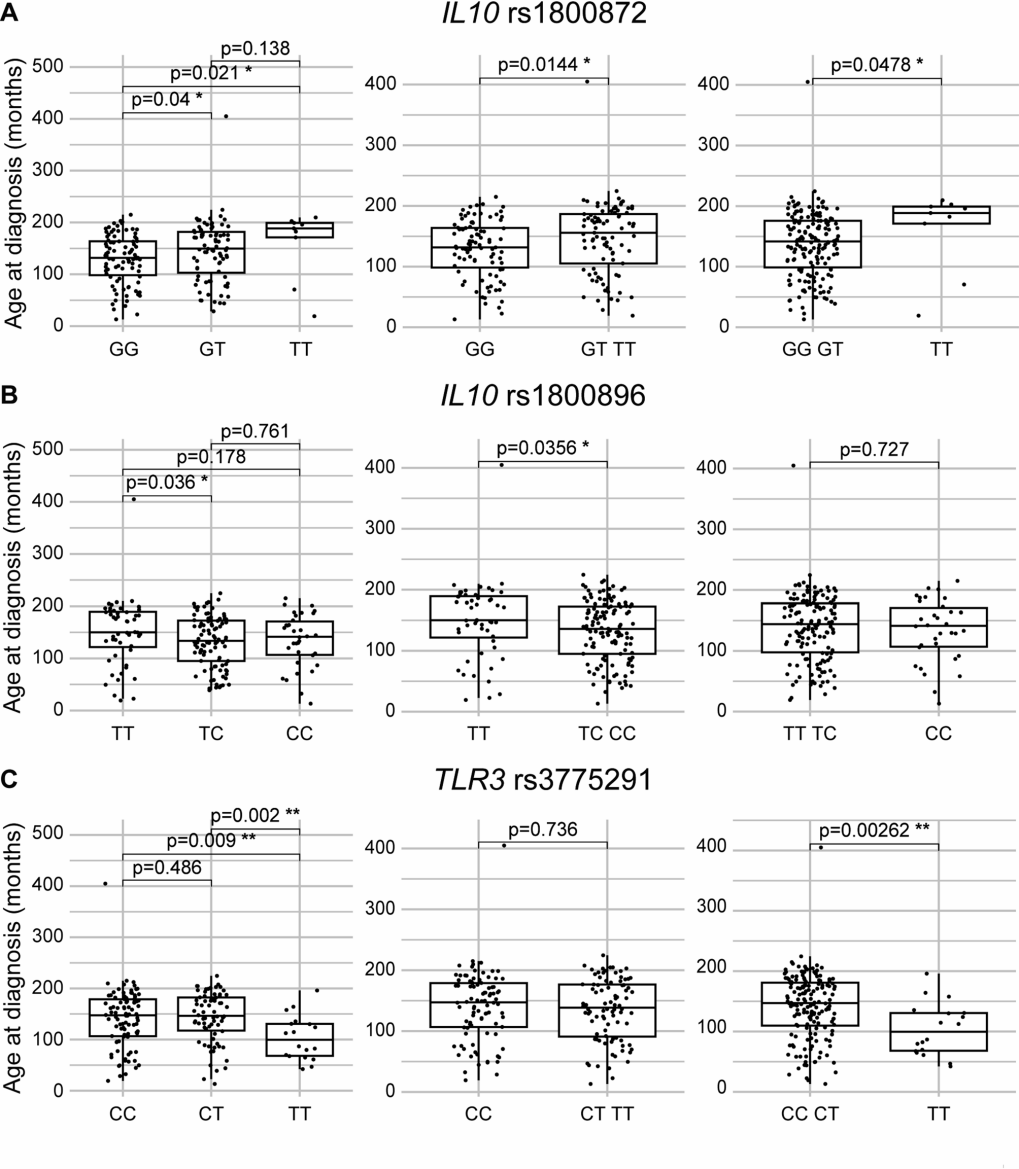
Age at diagnosis according to *IL10* and *TLR3* SNPs. Boxplots displaying median and interquartile ranges of ALK-positive ALCL patients age at diagnosis according to the genotype of selected SNPs. rs1800872 is shown in (**A**), rs1800896 in (**B**) and rs3775291 in (**C**). Dominant or recessive behavior of the variant allele was tested by grouping patients bearing at least one variant or reference allele (middle and right panels). Mann-Whitney test was used to calculate statistical significance, expressed as p values and *. *p < 0.05, **p < 0.01

Patient age at diagnosis was evaluated according to the genotype at fourteen SNP loci and three out of 14 SNPs were significantly associated with age at diagnosis: *IL10* rs1800872, *IL10* rs1800896 and *TLR3* rs3775291 (Fig. 1). The presence of at least one T nucleotide at rs1800872 locus associated with late disease onset (*p* = 0.0144). Median age at diagnosis of patients with the GG genotype was 131 months (CI 95% 123–142), whereas it increased to 150 (CI 95% 135–171) and 188 months (CI 95% 70–199) for patients bearing the GT and TT genotypes, respectively. Rs1800896 was also significantly associated with age at diagnosis. Notably, patients bearing at least one C presented with earlier onset than patients with TT genotype (*p* = 0.0356). Patients bearing SNP variant TT were diagnosed at 150 months (CI 95% 139–178) of age, whereas median diagnosis occurred at 133 (CI 95% 122–153) and 141 months (CI 95% 120–160) in patients bearing TC and CC variants, respectively. Finally, SNP rs3775291 of *TLR3* gene also associated with ALCL occurrence. The presence of a T nucleotide in homozygosity was associated with early diagnosis (99 months CI 95% 68–130). Diagnosis was significantly accelerated by 47 months as compared to the CT genotype (146 months CI 95% 134–168, *p* = 0.002) and as compared to the more frequent CC genotype (146 months CI 95% 130–161, *p* = 0.009).

### Association of polymorphisms with PFS

**Fig. 2:**
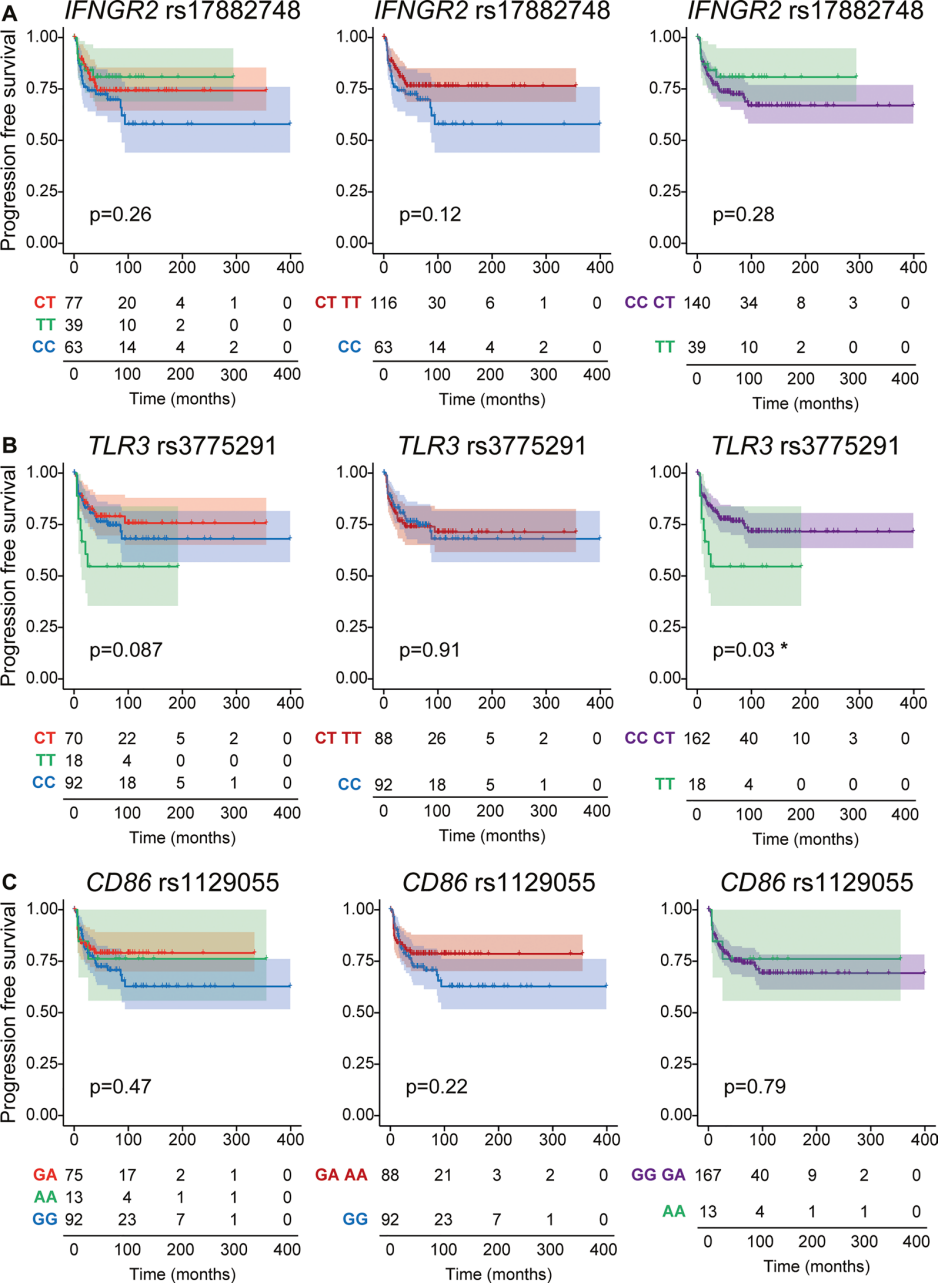
Progression-free survival of ALK-positive ALCL-patients according to selected SNP genotypes. Kaplan-Meier plots displaying progression-free survival of patients with ALK-positive ALCL after chemotherapy. Patients were categorized according to selected SNP genotypes. *IFNGR2* rs17882748 is shown in (**A**), *TLR3* rs3775291 in (**B**) and *CD86* rs1129055 in (**C**). Dominant or recessive behavior of the variant allele was tested by grouping patients bearing at least one variant or reference allele (middle and right panels). Observations of patients alive without failure were censored at the time of their last follow-up and indicated by a cross on the curve at the censoring time. Log-rank test was used to calculate statistical significance, expressed as p values and *. *p < 0.05. Numbers at the bottom of the figure are number at risk

**Fig. 3:**
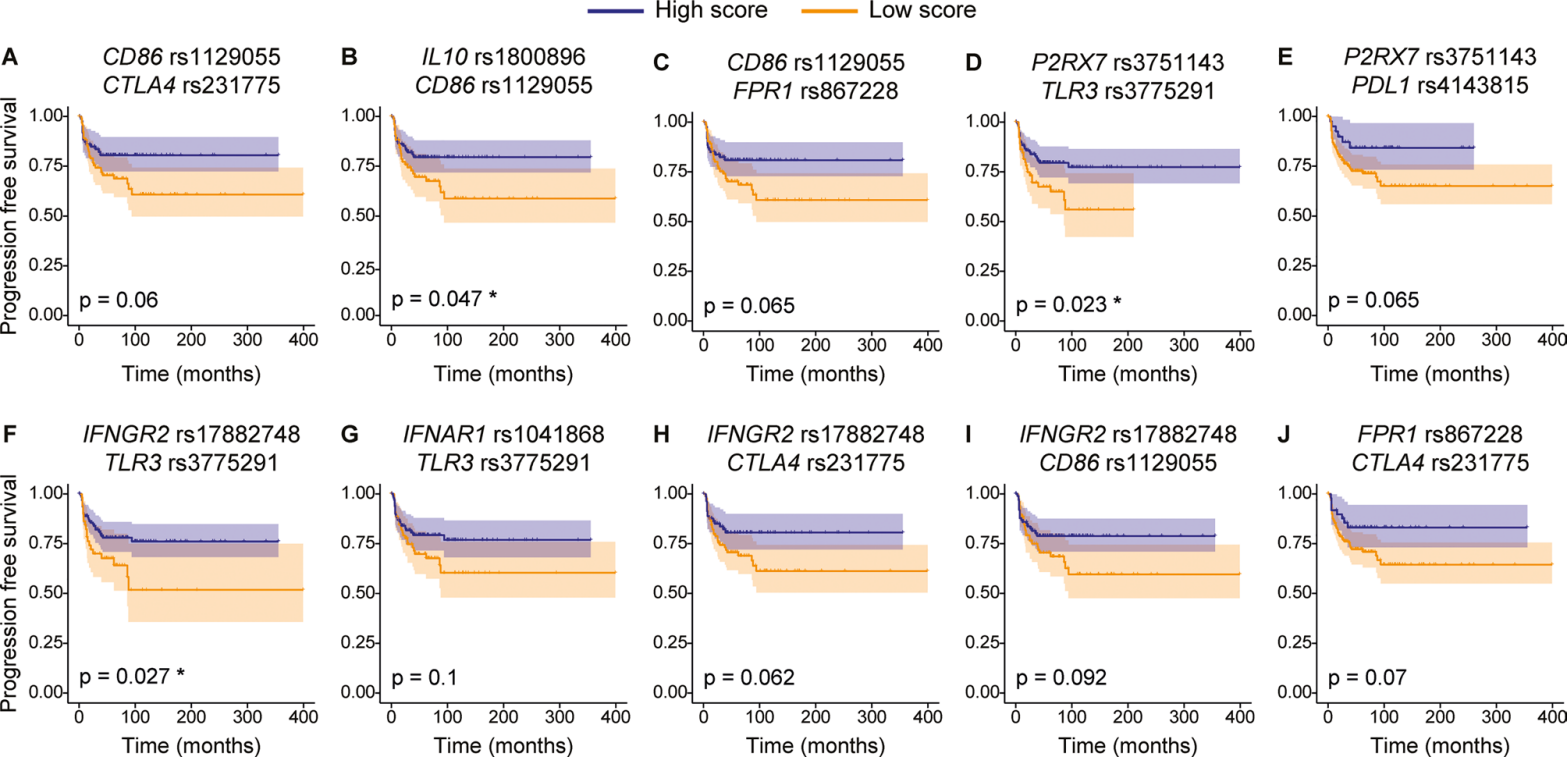
Progression-free survival of ALK-positive ALCL-patients according to selected combination of SNPs. Kaplan-Meier plots displaying progression-free survival of patients with ALK-positive ALCL after chemotherapy. Patients were categorized according to selected combination of SNPs after scoring, rs1129055 and rs231775 (**A**), rs1800896 and rs1129055 (**B**), rs1129055 and rs867228 (**C**), rs3751143 and rs3775291 (**D**), rs3751143 and rs4143815 (**E**), rs17882748 and rs3775291 (**F**), rs1041868 and rs3775291 (**G**), rs17882748 and rs231775 (**H**), rs17882748 and rs1129055 (**I**), rs867228 and rs231775 (**J**). Observations of patients alive without failure were censored at the time of their last follow-up and indicated by a cross on the curve at the censoring time. Log-rank test was used to calculate statistical significance, expressed as p values and *. *p < 0.05.

At the time of analysis 46 out of 180 patients enrolled in this study experienced a relapse event. Association of genetic polymorphisms with PFS suggested trends for *IFNGR2* rs17882748, *TLR3* rs3775291 and *CD86* rs1129055 to segregate patients with high risk from those who had low risk of relapsing after therapy (Fig. 2). *TLR3* rs3775291 seemed to associate with the risk of tumor recurrence in a recessive model. Patients bearing the T nucleotide in homozygosity had a statistically significantly higher risk of relapsing after therapy when compared to those having CC and CT at rs3775291 locus (TT vs. CT + CC *p* = 0.03). No other association between single SNPs and PFS was detected (Figure S1). We next evaluated whether the combination of multiple genetic variations correlated with PFS. Thus, we generated a score for each patient based on his or her polymorphism profile. The presence of alleles associated with higher PFS received higher score. Of all possible SNP combinations, ten of them tended to stratify patients in two groups with different risks of disease recurrence after chemotherapy (Fig. 3). Interestingly, the combination of SNP rs1129055 in *CD86* and rs231775 in *CTLA4* appeared to correlate with PFS as did the combination of rs1129055 and rs1800896 in *IL10*. Noteworthy, rs1129055 of *CD86* recurred in three different combinations.

### Association of polymorphisms with histologic subtype and anti-ALK antibody titers

The World Health Organization (WHO) recognizes five different subtypes of ALK-positive ALCL: common pattern, small cell pattern, lymphohistiocytic pattern, Hodgkin’s-like pattern and composite pattern. The small cell pattern (SC) and lymphohistiocytic subtype (LH) are independent risk factors determining a higher risk of relapsing compared to other subtypes [48, 49].

Reference histology was available for 96 patients. Thirty patients had ALCL with SC or LH subtypes and 66 ALCL with other patterns. Genotype frequency of the 14 cancer-immunity relevant SNPs according to the histological subtype is shown in Table 4. Genotypes were similarly distributed among SC/LH and other patterns (Table 4).

**Table 4:** Association of SNP genotype with histologic subtype of ALK-positive ALCL and circulating anti-ALK antibody level

		Histological subtype			Anti-ALK antibody level		
		Other subtypes n/tot (%)	SC/LH n/tot (%)	Fisher’s Exact Test *p* value	Low titern/tot(%)	High titer n/tot (%)	Fisher’s Exact Test *p* value
*CD274* rs4143815	GG	39/96 (40.6)	17/96 (17.7)	0.5937	11/80 (13.8)	39/80 (48.8)	0.4743
	GC	19/96 (19.8)	11/96 (11.5)		7/80 (8.7)	12/80 (15)	
	CC	8/96 (8.3)	2/96 (2.1)		3/80 (3.7)	8/80 (10)	
*CD86* rs1129055	GG	33/96 (34.4)	19/96 (19.8)	0.4013	11/80 (13.8)	29/80 (36.2)	0.6412
	GA	29/96 (30.2)	9/96 (9.4)		8/80 (10)	27/80 (33.8)	
	AA	4/96 (4.2)	2/96 (2.1)		2/80 (2.5)	3/80 (3.7)	
*CTLA4* rs231775	AA	31/96 (32.3)	15/96 (15.6)	0.8251	12/80 (15)	27/80 (33.8)	0.1604
	AG	32/96 (33.3)	13/96 (13.5)		9/80 (11.2)	23/80 (28.8)	
	GG	3/96 (3.1)	2/96 (2.1)		0/80 (0)	9/80 (11.2)	
*ECE1* rs867228	GG	59/96 (61.5)	29/96 (30.2)	0.4283	18/80 (22.5)	50/80 (62.5)	1
	GA	7/96 (7.3)	1/96 (1)		3/80 (3.8)	9/80 (11.2)	
*FPR1* rs867228	GG	39/96 (40.6)	21/96 (21.9)	0.6267	11/80 (13.8)	37/80 (46.2)	0.2784
	GT	24/96 (25)	8/96 (8.3)		10/80 (12.5)	18/80 (22.5)	
	TT	3/96 (3.1)	1/96 (1)		0/80 (0)	4/80 (5)	
*IFNAR1* rs1041868	GG	44/96 (45.8)	21/96 (21.9)	0.5642	16/80 (20)	37/80 (46.2)	0.5277
	GA	18/96 (18.7)	9/96 (9.4)		5/80 (6.3)	20/80 (25)	
	AA	4/96 (4.2)	0/96		0/80 (0)	2/80 (2.5)	
*IFNGR2* rs17882748	CC	21/96 (21.9)	12/96 (12.5)	0.6866	10/79 (12.7)	17/79 (21.5)	0.2863
	CT	30/96 (31.3)	13/96 (13.5)		8/79 (10.1)	26/79 (32.9)	
	TT	15/96 (15.6)	5/96 (5.2)		3/79 (3.8)	15/79 (19)	
*IL10* rs1800872	GG	30/96 (31.2)	16/96 (16.7)	0.4573	12/80 (15)	30/80 (37.5)	0.4992
	GT	32/96 (33.3)	14/96 (14.6)		9/80 (11.2)	24/80 (30)	
	TT	4/96 (4.2)	0/96		0/80 (0)	5/80 (6.3)	
*IL10* rs1800896	TT	19/96 (19.8)	6/96 (6.2)	0.6901	8/80 (10)	17/80 (21.2)	0.6232
	TC	34/96 (35.4)	18/96 (18.8)		11/80 (13.8)	31/80 (38.8)	
	CC	13/96 (13.5)	6/96 (6.2)		2/80 (2.5)	11/80 (13.7)	
*PDCD1* rs2227981	GG	12/96 (12.5)	9/96 (9.4)	0.2144	7/80 (8.8)	16/80 (20)	0.9425
	GA	45/96 (46.9)	15/96 (15.6)		10/80 (12.5)	31/80 (38.7)	
	AA	9/96 (9.4)	6/96 (6.2)		4/80 (5)	12/80 (15)	
*P2RX7* rs3751143	AA	34/96 (35.4)	21/96 (21.9)	0.2314	9/80 (11.2)	37/80 (46.2)	0.2049
	AC	30/96 (31.2)	9/96 (9.4)		12/80 (15)	21/80 (26.3)	
	CC	2/96 (2.1)	0/96		0/80	1/80 (1.3)	
*TGFB1* rs1800469	GG	30/96 (31.3)	15/96 (15.6)	0.6622	9/80 (11.2)	28/80 (35)	0.948
	GA	28/96 (29.2)	10/96 (10.4)		9/80 (11.3)	22/80 (27.5)	
	AA	8/96 (8.3)	5/96 (5.2)		3/80 (3.8)	9/80 (11.2)	
*TLR3* rs3775291	CC	31/96 (32.3)	16/96 (16.6)	0.7301	12/80 (15)	31/80 (38.7)	0.2584
	CT	28/96 (29.2)	10/96 (10.4)		6/80 (7.5)	25/80 (31.2)	
	TT	7/96 (7.3)	4/96 (4.2)		3/80 (3.8)	3/80 (3.8)	
*TLR4* rs4986790	AA	60/96 (62.5)	27/96 (28.1)	1	21/80 (26.2)	54/80 (67.5)	0.3184
	AG	6/96 (6.3)	3/96 (3.1)		0/80	5/80 (6.3)	

Anti-ALK antibody titer in serum or plasma is a surrogate marker of the strength of a specific anti-tumor immune response orchestrated by T lymphocytes and mediated by B cells. Low anti-ALK antibody titer at diagnosis have been shown to correlate with high relapse risk [5, 6]. We investigated whether SNPs of the 13 cancer-immunity relevant genes would associate with the anti-ALK antibody titer of a subset of patients (*n* = 80). None of the SNPs tested correlated to the titer when grouped as previously published (Table 4).

### Association of polymorphisms with TLR3 and IL10 gene expression

**Fig. 4:**
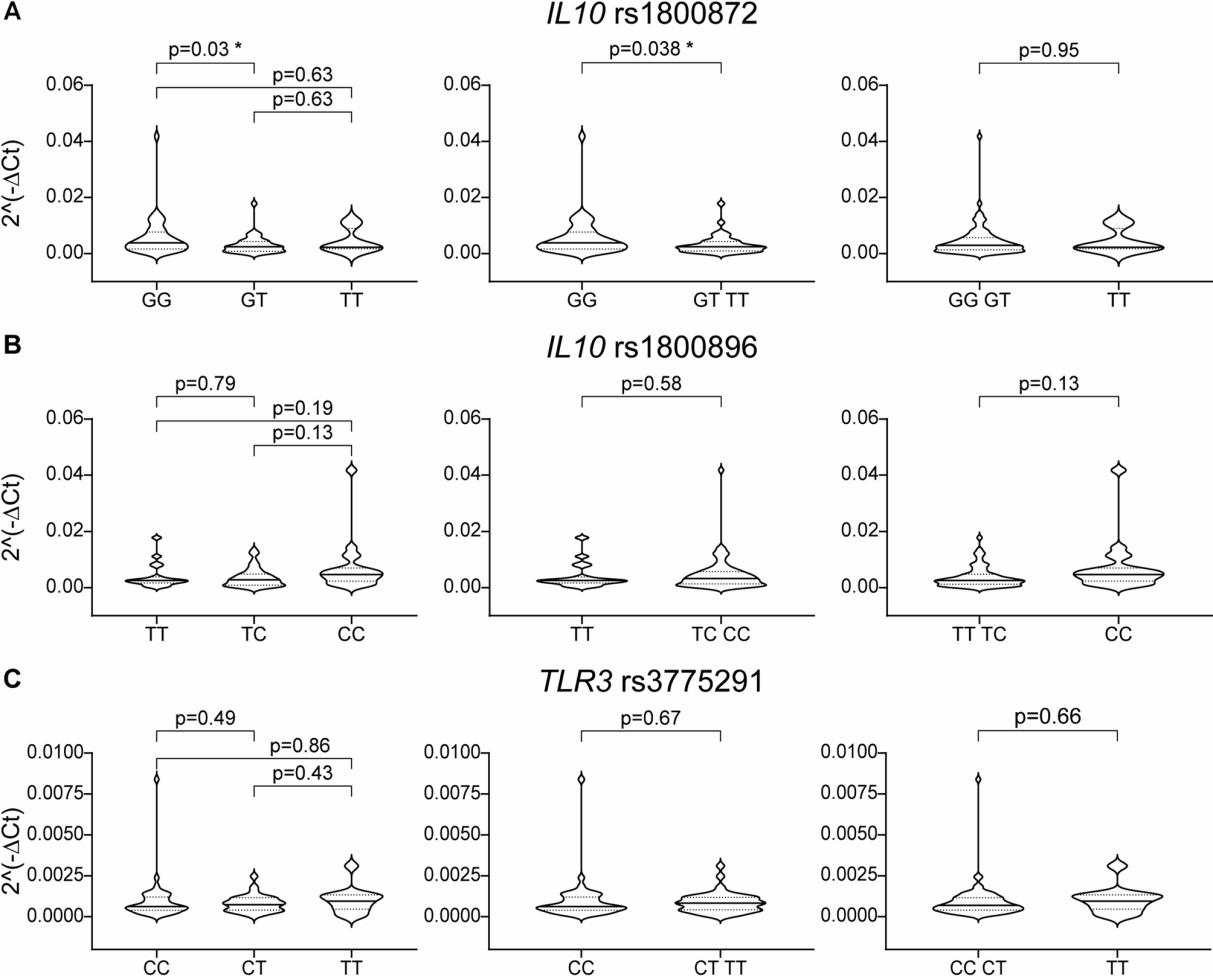
*IL10* and *TLR3* expression levels according to rs1800872, rs1800896 and rs3775291 genotypes. *IL10* and *TLR3* mRNA levels in circulating PBMCs of ALK-positive ALCL patients (*n* = 85) were assessed using qRT-PCR. Relative expression, as 2^(−ΔCt), is presented according to rs1800872 (**A**), rs1800896 (**B**) or rs3775291 (**C**) genotypes. Statistical significance was calculated using the Student’s t-test with Welch’s correction and expressed as p values and *. *p < 0.05, **p < 0.01

**Fig. 5:**
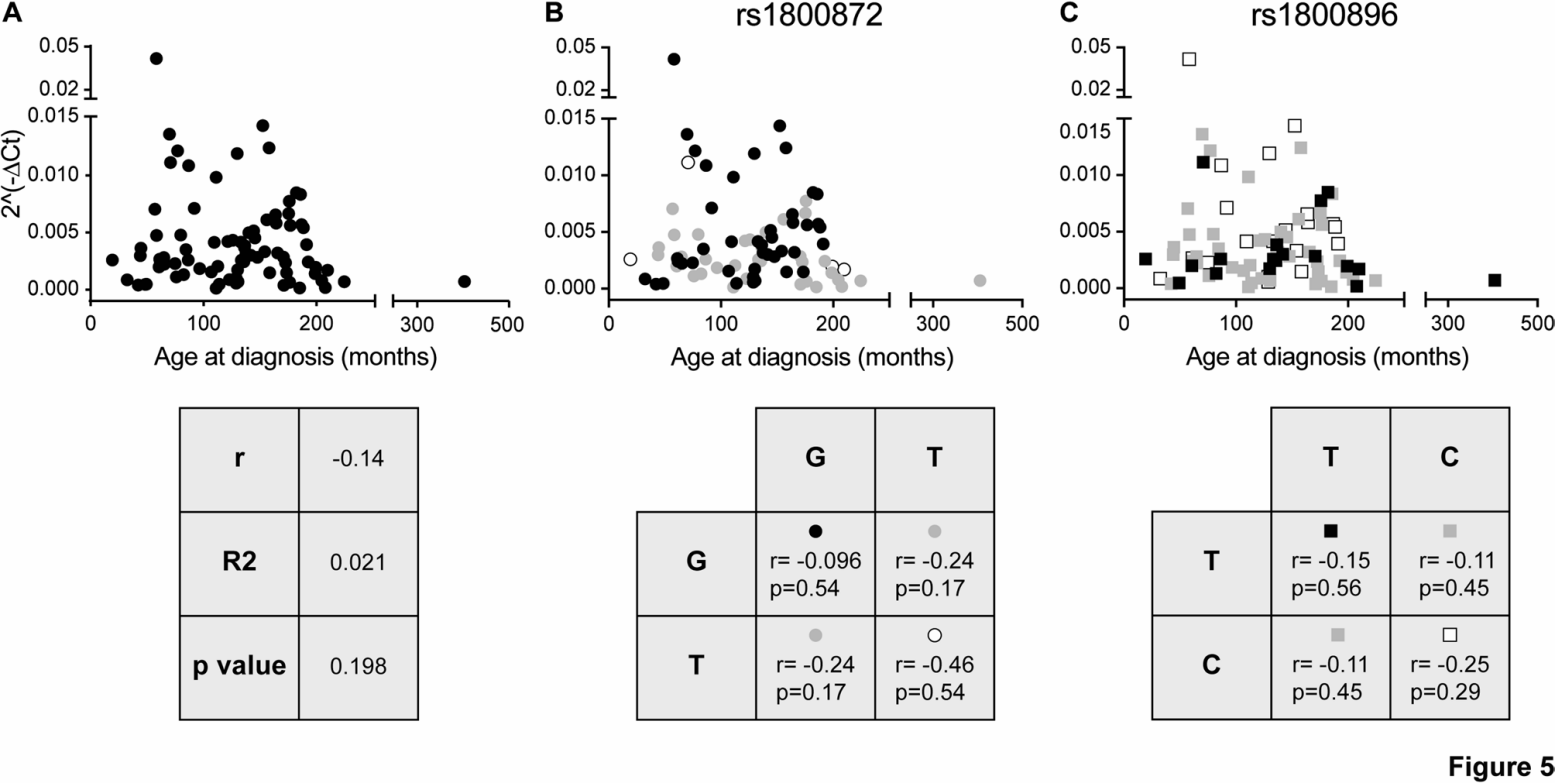
IL10 mRNA levels, expressed as 2^(−ΔCt), of ALK-positive ALCL patients (*n* = 82) are shown against patients age at diagnosis, expressed in months (**A**). Correlation between IL10 expression level and age at diagnosis according to SNP genotype of rs1800872 (**B**) and rs1800896 (**C**). Pearson’s correlation coefficients and statistical significance are shown in the box below the respective graph

Single nucleotide variations may affect gene function or expression depending on their location. Upstream variants alter the promoter sequences of genes, thus regulating their transcription and the abundance of mRNAs and proteins. Alternatively, SNPs located in exons may modify the protein sequence, thus altering the structure, stability and function. We wondered whether TLR3 or IL10 gene expression would be affected by rs3775291 or rs1800872/rs1800896 genotypes, respectively. To address this question, we quantified TLR3 and IL10 transcripts in PBMCs isolated from 85 patients and bearing the three alternative variants of the SNPs. The rs3775291 genotype had no impact on TLR3 RNA levels. However, both rs1800872 and rs1800896 modestly correlated with IL10 gene expression, as expected by SNP location in the promoter region (Table 2). The presence of at least one T at rs1800872 was associated with a lower median of IL10 expression as compared to the GG genotype (GT TT vs. GG fold change = 0.63, *p* = 0.038). Similarly, patients bearing the CC allele at rs1800896 showed a trend for higher IL10 expression (CC vs. TT TC fold change = 1.8, *p* = 0.13) (Fig. 4). Linear correlation between age at diagnosis and IL10 mRNA levels did not reach statistical significance due to the limited number of samples (Fig. 5). However, even if not statistically significant, IL10 expression tended to negatively correlate with age at diagnosis for patients heterozygous at rs1800872 site (Fig. 5). The subgroup of heterozygous patients experiencing later diagnosis were having lower level of IL-10 mRNA.

### Expression profile of immune-related genes by ALK-positive ALCL tumor

**Fig. 6:**
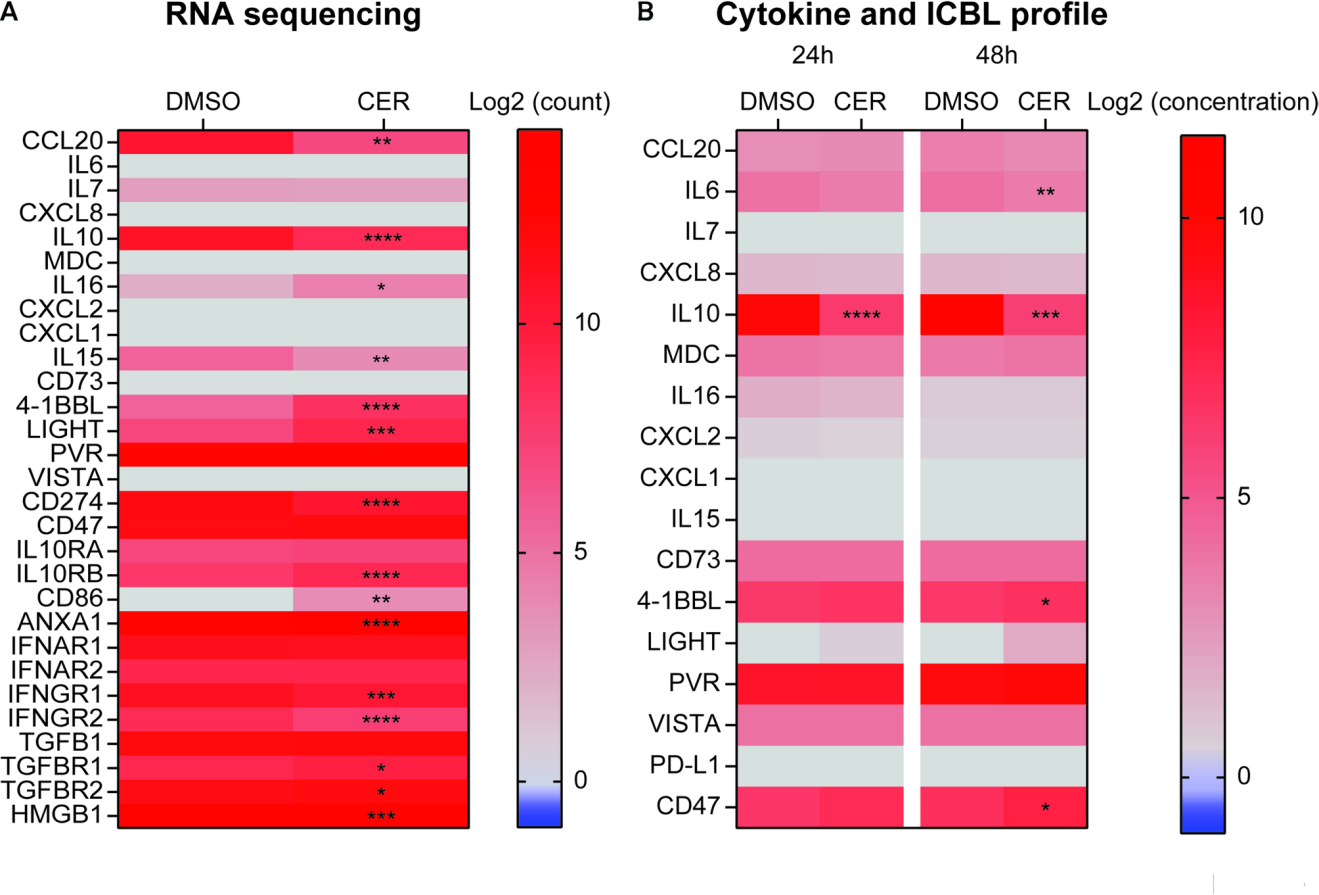
Transcriptome and secretome of ALK-positive ALCL cell line SU-DHL-1. Transcriptomic profile of selected mRNAs of human NPM1::ALK-positive SU-DHL-1 cells treated with the ALK inhibitor ceritinib (50nM) for 24 h. The expression level of immune-relevant transcripts of ceritinib-treated cells were compared to vehicle-treated cells and fold changes (in logarithmic scale) are depicted (**A**). Secretion profile of selected cytokines and immune-checkpoint ligands of human NPM1::ALK-positive SU-DHL-1 cells treated with the ALK inhibitor ceritinib (50nM) for 24–48 h. Cytokine and immune checkpoint ligands levels of ceritinib-treated cells were compared to vehicle-treated cells and fold changes (in logarithmic scale) are depicted (**B**). Statistical significance was calculated using the multiple Student’s t-test. *p ≤ 0.05, **p ≤ 0.01, ***p ≤ 0.001, ****p ≤ 0.0001

Next, we characterized the expression of immune-relevant genes in an ALK-positive ALCL cell line, namely SU-DHL-1. We took advantage of the treatment with ceritinib, second generation ALK inhibitor, to reveal any ALK-dependent effect. For that purpose, we sequenced SU-DHL-1 transcriptome upon treatment with ceritinib and measured secretion of a panel of 20 cytokines and immune-related ligands after ALK inhibition (Fig. 6). Notably, SU-DHL-1 expressed many immune-related transcripts in an ALK-dependent manner, because expression significantly decreased after ceritinib treatment (*CCL20*, *IL10*, *IL15*, *CD274* or *IFNGR*). On the contrary, ALK kinase activity repressed the expression of *CD86* and the B subunit of IL10 receptor (*IL10RB*), because evident expression was found after ALK inhibition. Expression at the mRNA level paralleled with protein expression and/or secretion for some of the cytokines and immune-related ligands analyzed, namely IL10 (Fig. 6).

It appears that ALK-positive ALCL tumor, or at least a representative cell line, presents many receptors and/or ligands, which participate in the anti-tumor immune response and possibly skew it toward its growth.

## Discussion

Many lines of evidence indicate that the anti-tumor immune response, which develops naturally or after immunogenic therapy, contributes to eradicate ALK-positive ALCL [4, 5, 7–9, 50]. With the aim of deepening the knowledge of immune-based mechanisms that might influence tumor onset and relapse in ALK-positive ALCL patients, we assessed 14 genetic variants of 13 genes potentially involved in the anti-tumor immune response in 180 patients. First, we found that age at diagnosis differed among patients carrying genetic variants of TLR3 and IL10, suggesting a role of these pathways in the spontaneous, therapy-independent anti-tumor immune response. TLR3 contributes to the activation of the innate immune response [51, 52] and the substitution C >T at rs3775291 leads to an amino acid change, which reduces TLR3 dimerization and RNA binding capacity, thus compromising downstream signaling [53]. Interestingly, ALK-positive ALCL patients bearing TT at rs3775291 were diagnosed at a comparatively earlier age. Notably, TLR3 has also a critical role in immunogenic therapy-induced immune response in breast cancer patients [23, 24]. ALCL patients included in our study were treated with a chemotherapy regimen, which included doxorubicin, a very well-known immunogenic cell death-inducing agent [10, 54]. TLR3 genetic polymorphism also showed a trend to segregate patients with high risk from those who had low risk of relapsing after therapy, and the presence of the loss-of function allele (T) in homozygosity, which occurs in 8% of the global population, tended to correlate with a higher risk of tumor recurrence.

Our analysis also suggested with caution that malignant disease was diagnosed later in patients with reduced expression of IL10 mRNA. Noteworthy, patients bearing at least one T at rs1800872 (which occurs in 29% of the general population), were diagnosed relatively late and presented with lower IL10 mRNA expression. Although transcript levels do not necessarily reflect protein levels and further confirmation in a larger cohort of patients is needed, it is tempting to speculate that high IL10 levels, associated with unfavorable SNPs, suppressed the spontaneous anti-tumor immune response and hence accelerated the development of ALK-positive ALCL. Notably, IL10 may also have a direct effect on ALK-positive ALCL tumor cells because the ALCL cell line, SU-DHL1, expresses IL10 receptor subunits A and B in an ALK-dependent fashion (Fig. 6). In these cells, activation of IL10 receptor signaling triggers STAT3 phosphorylation, which in turn promotes tumor cell survival and proliferation. Moreover, transfection-enforced overexpression of IL10RA confers resistance to ALK inhibitors [55].

Finally, we assessed whether certain combinations of SNPs were associated with patient prognosis. We uncovered several combinations that showed a trend to predict prognosis, although such effects must be confirmed in a larger cohort of patients. However, the combination of two SNPs, namely rs1129055 of *CD86* gene and rs231775 of the *CTLA4* gene caught our attention because CD86 and CTLA4 engage in a receptor-ligand interaction that inhibit anti-tumor immune response [56]. The G >A transition in *CD86* gene (rs1129055), which occurs in 27% of the global population (at least at one allele), introduces a phosphorylation site in the cytoplasmic region of CD86, which alters its downstream signals [57]. Patients with kidney transplantation harboring AA genotype exhibited a reduced risk of acute rejection, suggesting a reduced level of immune activation [58]. Similarly, the A>G transition at rs231775 affecting *CTLA4* causes an amino acid substitution in CTLA4 protein that reduces the expression levels of the receptor. Accordingly, the rs231775 GG genotype, occurring in 13.8% of the general population, is associated with higher T cell activation and proliferation and, in fact, is more frequent in patients with autoimmune disorders, such as rheumatoid arthritis and Hashimoto thyroiditis [59–62]. Our results suggested that the simultaneous presence of GG at rs1129055 and at least one A at rs231775 tended to correlate with a higher risk of post-therapeutic relapse. These results seem to be, at least in part, in contradiction with the assumption that higher T cell activation corresponds to successful tumor control. However, anti-tumor immune response becomes efficient because of the fine tuning of co-stimulatory and inhibitory signals. Of note, excess of inhibitory signals may favor tumor escape. Moreover, when inflammatory or co-stimulatory signals prevail, tumor cell proliferation may predominate, or autoimmunity arise as unwanted side effect. The discrepancy between trends shown by our results and known effect of rs1129055 on immune system activation may be also explained by the expression of CD86 by ALK-positive ALCL tumor cells. SU-DHL-1 expressed CD86, at the mRNA level (Fig. 6), and CD86 of tumor origin might intervene to alter the thin equilibrium that decides T cell activation threshold.

## Limitation of the study

We believe that our findings reinforce the clinical relevance of immunosurveillance in the ultimate control of ALK-positive ALCL and support a potential contribution of the IL10 and TLR3 pathways to its pathogenesis. Nevertheless, certain limitations should be acknowledged. First, because ALCL is a rare disease with an incidence of approximately 1.2 cases per million persons per year, our patient cohort was small, and the study may be underpowered to detect the effect of individual single-nucleotide variants on disease presentation and progression. Therefore, validation in larger patient cohorts is essential before our observations can be considered definitive. Moreover, while our data suggest a role for the IL10 and TLR3 pathways in ALK-positive ALCL pathogenesis, these associations do not establish causality. Further mechanistic studies are required to substantiate the potential pro-ALCL effects of IL10 and TLR3.

## Conclusion

To sum up, despite its limitations, our study further supports the importance of immunosurveillance in the ultimate control of ALK-positive ALCL. Consistent with this notion, genetic variants influencing the threshold of immune activation were found to be associated with patient age at diagnosis and risk of relapse, suggesting that the IL10 and TLR3 pathways contribute to the pathogenesis of ALK-positive ALCL.

## Electronic Supplementary Material

Below is the link to the electronic supplementary material.

Supplementary Material 1

Supplementary Material 2

## Data Availability

The datasets used and/or analysed during the current study are available from the corresponding author on reasonable request.

## Abbreviations

ALCL: Anaplastic large cell lymphoma
ALK: Anaplastic lymphoma kinase
CER: Ceritinib
EML4: Echinoderm microtubule-associated protein-like
FBS: Fetal bovine serum
HLA-DRB1: Human leukocyte antigen DR beta 1 chain
ICL: Immune checkpoint ligands
LH: Lymphohistiocytic subtype
MDD: Minimal disseminated disease
NPM1: Nucleophosmin 1
PBMCs: Peripheral blood mononuclear cells
Pen/Strep: Penicillin/streptomycin
PD-1: Programmed cell death protein 1
PFS: Progression-free survival
RIN: RNA integrity number
SC: Small cell pattern
SNP: Single nucleotide polymorphism
TPM3: Tropomyosin 3
WHO: World Health Organization
