# Supplementary figures and images for: Association of immune relevant single nucleotide polymorphisms with ALK-positive anaplastic large cell lymphoma presentation and outcome: results of the immuno ALCL study
Source: J Transl Med. 2025 Dec 30;23:1434. doi: 10.1186/s12967-025-07410-5 (PMC12754919; doi:10.1186/s12967-025-07410-5)

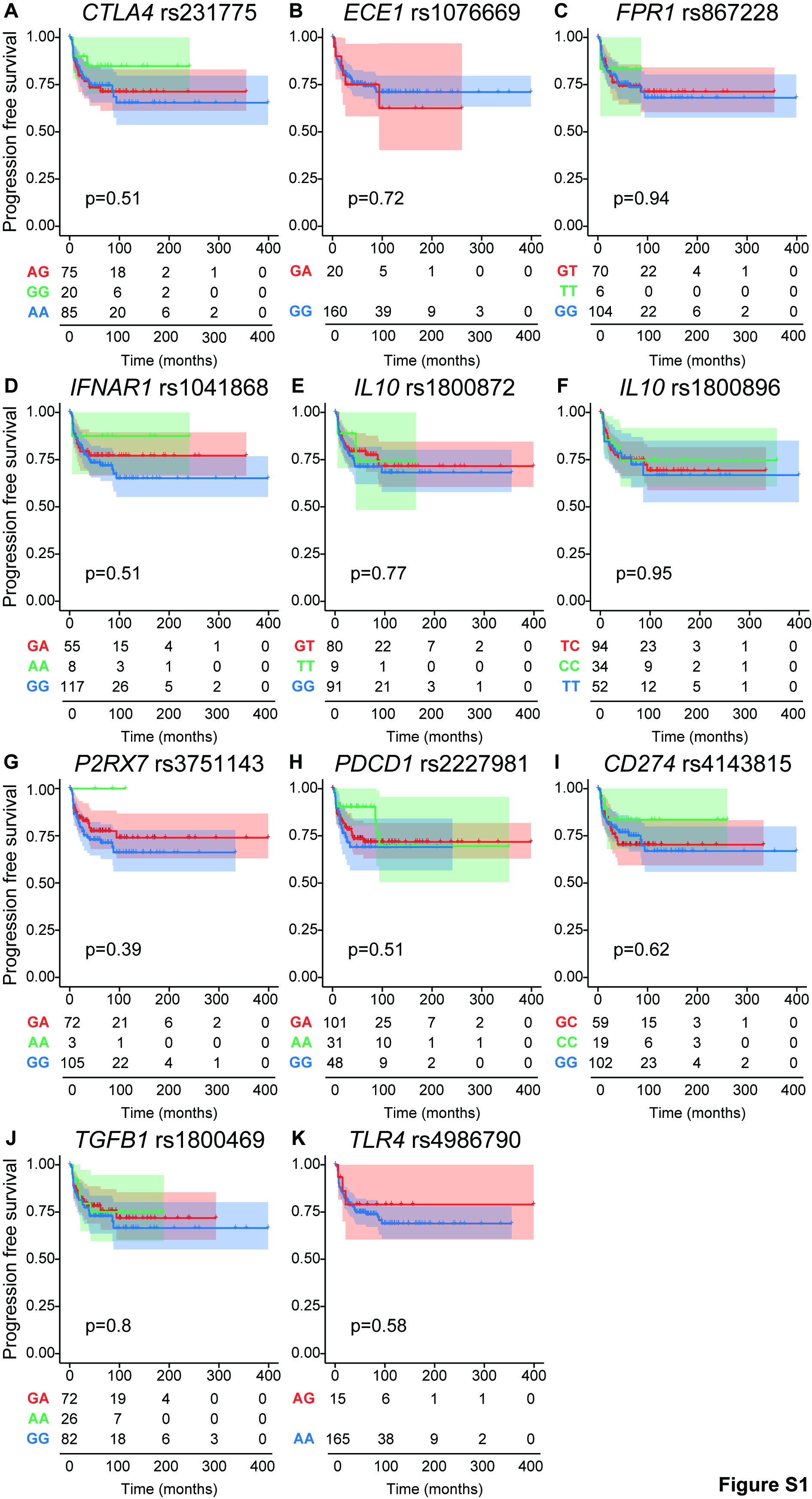

Supplement: Supplementary file 1 — Supplementary Material 1 [file 12967_2025_7410_MOESM1_ESM.tif]

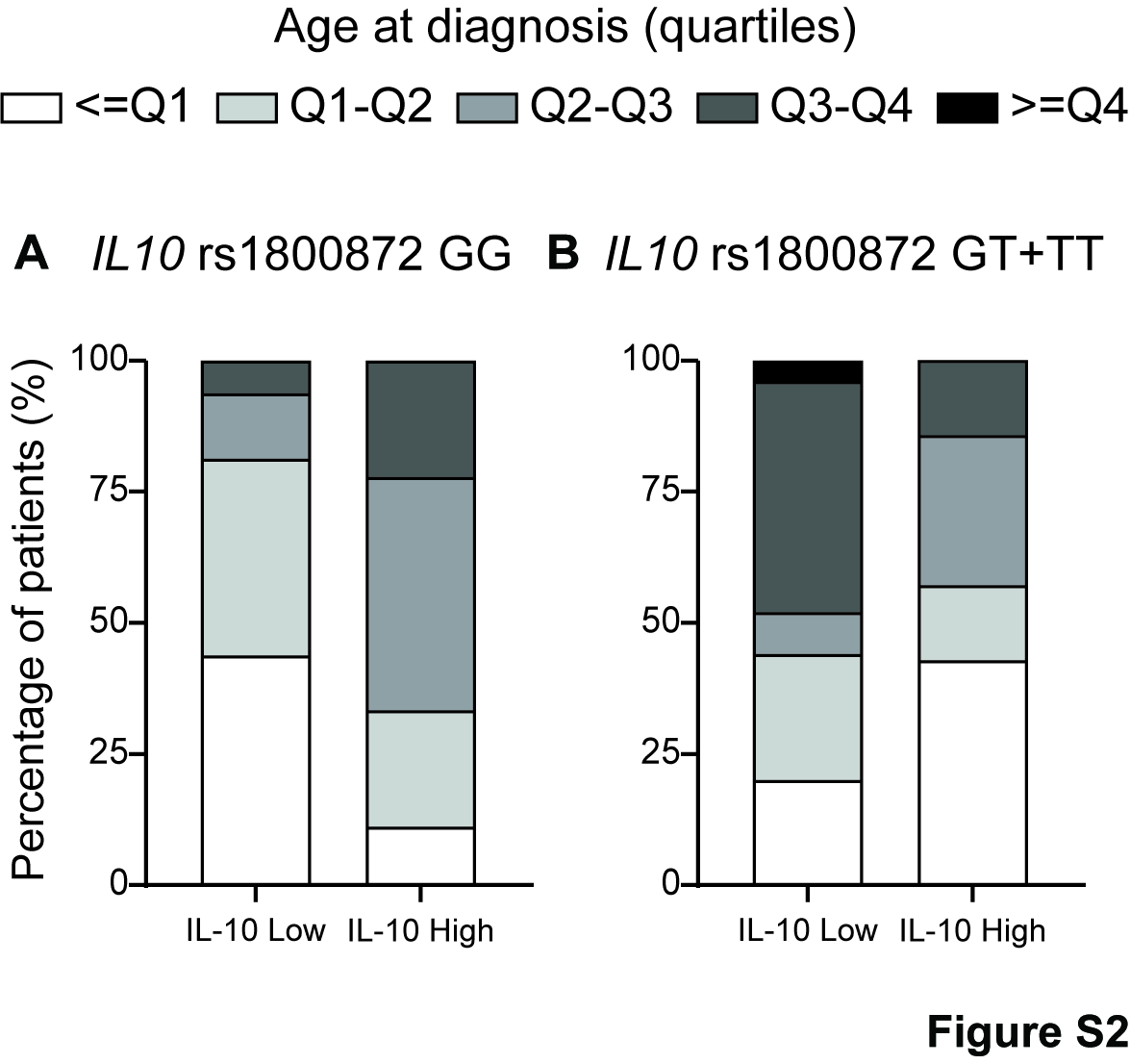

Supplement: Supplementary file 2 — Supplementary Material 2 [file 12967_2025_7410_MOESM2_ESM.tif]
